# Supplementary material for: Altered expression of a quality control protease in E. coli reshapes the in vivo mutational landscape of a model enzyme
Source: eLife. 2020 Jul 23;9:e53476. doi: 10.7554/eLife.53476 (PMC7377907; doi:10.7554/eLife.53476)
Supplement: Transparent reporting form [file elife-53476-transrepform.docx]

***eLife’s* transparent reporting form**

We encourage authors to provide detailed information *within their submission* to facilitate the interpretation and replication of experiments. Authors can upload supporting documentation to indicate the use of appropriate reporting guidelines for health-related research (see [EQUATOR Network](http://www.equator-network.org/%20)), life science research (see the [BioSharing Information Resource](https://biosharing.org/)), or the [ARRIVE guidelines](http://www.plosbiology.org/article/info:doi/10.1371/journal.pbio.1000412) for reporting work involving animal research. Where applicable, authors should refer to any relevant reporting standards documents in this form.

If you have any questions, please consult our Journal Policies and/or contact us: [editorial@elifesciences.org](mailto:editorial@elifesciences.org).

**Sample-size estimation**

- You should state whether an appropriate sample size was computed when the study was being designed
- You should state the statistical method of sample size computation and any required assumptions
- If no explicit power analysis was used, you should describe how you decided what sample (replicate) size (number) to use

Please outline where this information can be found within the submission (e.g., sections or figure legends), or explain why this information doesn’t apply to your submission:

No samples sizes were calculated, and no explicit power analysis was used. Sample size is not a consideration for this study. We consider all possible single amino acids substitutions in DHFR; information on replicates is given throughout the manuscript in Results, Methods, and in Figure legends.

**Replicates**

- You should report how often each experiment was performed
- You should include a definition of biological versus technical replication
- The data obtained should be provided and sufficient information should be provided to indicate the number of independent biological and/or technical replicates
- If you encountered any outliers, you should describe how these were handled
- Criteria for exclusion/inclusion of data should be clearly stated
- High-throughput sequence data should be uploaded before submission, with a private link for reviewers provided (these are available from both GEO and ArrayExpress)

Please outline where this information can be found within the submission (e.g., sections or figure legends), or explain why this information doesn’t apply to your submission:

Information on replicates, their definition, and errors calculated from replicates are clearly described in the Figure legends, Methods, Supplementary Tables and Source Data.

All experiments were performed in triplicate with the exception of thermal denaturation. For *in vivo* experiments, replicates were biological replicates starting from fresh transformations of plasmid library into *E. coli* competence cells generated from a single progenitor colony. For *in vitro* experiments, replicates were independent experiments starting from a fresh aliquot of frozen purified enzyme using aliquots from the same stock of buffer and fresh preparations of substrate.

Deep sequencing reads were filtered, allele counts were subjected to a threshold minimum, and selection coefficients with poor reproducibility were discarded as described in Methods. No other data were discarded.

Raw deep sequencing data have been uploaded to the NCBI Sequence Read Archive (SRA) as described in Data and materials availability.

**Statistical reporting**

- Statistical analysis methods should be described and justified
- Raw data should be presented in figures whenever informative to do so (typically when N per group is less than 10)
- For each experiment, you should identify the statistical tests used, exact values of N, definitions of center, methods of multiple test correction, and dispersion and precision measures (e.g., mean, median, SD, SEM, confidence intervals; and, for the major substantive results, a measure of effect size (e.g., Pearson's r, Cohen's d)
- Report exact p-values wherever possible alongside the summary statistics and 95% confidence intervals. These should be reported for all key questions and not only when the p-value is less than 0.05.

Please outline where this information can be found within the submission (e.g., sections or figure legends), or explain why this information doesn’t apply to your submission:

Statistical analyses are described in the Methods, and the mean and standard deviation of experimental replicates is reported in Figures, Figure legends, and Source Data and Supplementary files. The standard error from linear regression in the calculation of selection coefficients is as described in the manuscript reporting the Enrich2 computational method and software, and this citation is included in Methods. For deep sequencing, the distributions of these statistical values and the Pearson R^2^ values of biological replicates are reported in Figure 1 – figure supplement 3 and Figure 2 – figure supplement 1 and are described the corresponding figure legends.

No additional statistical methods are used.

(For large datasets, or papers with a very large number of statistical tests, you may upload a single table file with tests, Ns, etc., with reference to sections in the manuscript.)

**Group allocation**

- Indicate how samples were allocated into experimental groups (in the case of clinical studies, please specify allocation to treatment method); if randomization was used, please also state if restricted randomization was applied
- Indicate if masking was used during group allocation, data collection and/or data analysis

Please outline where this information can be found within the submission (e.g., sections or figure legends), or explain why this information doesn’t apply to your submission:

Group allocation is not applicable to this study because all samples were treated uniformly in each experiment.

**Additional data files (“source data”)**

- We encourage you to upload relevant additional data files, such as numerical data that are represented as a graph in a figure, or as a summary table
- Where provided, these should be in the most useful format, and they can be uploaded as “Source data” files linked to a main figure or table
- Include model definition files including the full list of parameters used
- Include code used for data analysis (e.g., R, MatLab)
- Avoid stating that data files are “available upon request”

Please indicate the figures or tables for which source data files have been provided:

Source data have been provided for the manuscript figures as described below.

Figure 1: Figure 1 – source data 1-3, Supplementary file 1

Figure 2: Supplementary files 1 and 2

Figure 3: Figure 3 – source data 1, Supplementary files 1 and 2

Figure 4: Figure 4 – source data 1-3, Supplementary file 7

Figure 5: Figure 3 – source data 1

Code for analysis is available in our GitHub repository for this project (<https://github.com/keleayon/2019_DHFR_Lon.git>) along with key input files and example command lines. This link is provided in Methods.

Raw deep sequencing data have been uploaded to the NCBI Sequence Read Archive (SRA) as described in Data and materials availability.

Allele counts used to generate the selection coefficients (all figures) are reported in Supplementary files 4-6.
